# How to encourage students in learning chemistry?

**DOI:** 10.1093/nsr/nwac267

**Published:** 2022-11-25

**Authors:** Weijie Zhao

**Affiliations:** Weijie Zhao is an NSR news editor based in Beijing

## Abstract

Chemistry is a basis for almost all science and engineering disciplines, and needs to be well mastered by both researchers and students. But in the eyes of many Chinese students and their parents, chemistry is not an initial choice because first, it seems to be less important than mathematics and physics, and second, chemistry-majored students usually cannot get high salaries after graduation, and may encounter safety risks when conducting chemical experiments.

How can we encourage students in learning chemistry? How can we improve higher chemistry education so that students can truly grasp the basic chemical principles, increase their chemical skills and apply the learnt knowledge in their future careers? Here in this panel discussion, six experts in higher chemistry education try to give their observations and opinions on these issues.

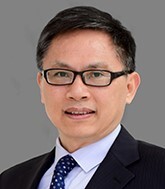

Yadong Li

President at Anhui Normal University; inorganic chemist.

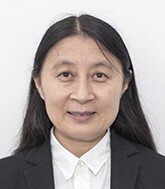

Yan Li

Professor at College of Chemistry and Molecular Engineering, Peking University.

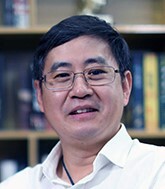

Jian Pei

Professor at College of Chemistry and Molecular Engineering, Peking University.

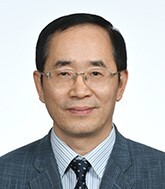

Zhenfeng Xi

Professor at College of Chemistry and Molecular Engineering, Peking University.

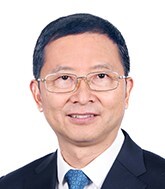

Song Gao (Chair)

President at Sun Yat-sen University; inorganic chemist.

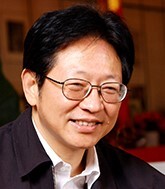

Lan-Sun Zheng (Chair)

Professor at College of Chemistry and Chemical Engineering, Xiamen University.

## CHEMISTRY SHOULD STAND IN THE SPOTLIGHT

**S Gao:** Chemistry is a basic science for most of the natural science disciplines. But its significance has not been fully emphasized in our educational system. If we broadly divide natural science into life science, which is organic, and physical science, which is inorganic, then chemistry is a basis for both of them. However, in the field of physical science, physics is universally considered to be a basis, leaving chemistry somewhat neglected.

**YD Li:** Our emphasis on chemistry lags much behind physics. In the national college entrance examination, a good physics score is required by almost all the science and engineering majors, but many majors do not require chemistry scores. In college, all science and engineering students need to learn general physics courses, but chemistry courses are not required by many majors. In practice, however, the development of many currently hot research directions—biomedicine, materials science, carbon emission and integrated circuit—are actually limited not by physics, but by chemistry. In industrial fields, many engineers and workers are not equipped with chemistry knowledge about hazardous materials, leading to potential safety risks.

**S Gao:** The public often links chemistry with pollution and safety accidents. With these misgivings, many students would not choose chemistry as their major. But we should tell them that if we want to solve these problems, the only way is to be equipped with knowledge of chemistry and promote research of green chemistry and sustainable chemistry.

**ZF Xi:** We need to inform the public that pollution and safety accidents are not results of chemistry, but results of human misbehaviors.

**YD Li:** Currently, students can be enrolled into Colleges of Chemistry by getting a relatively low score. I think this situation should be changed. If we enroll only the students with high scores in chemistry, as well as mathematics and physic, the students would begin to consider chemistry as a significant discipline.

In the field of physical science, physics is universally considered to be a basis, but chemistry is somewhat neglected.

—Song Gao

## CURRICULA REFORM IN THE NEW ERA OF INTERDISCIPLINARY RESEARCH

**S Gao:** Scientific disciplines are quickly cross-linking with each other, and chemistry is one of the centers of this network, linking with many disciplines such as life sciences, information sciences and materials science.

**ZF Xi:** Artificial intelligence (AI), robotics and many other technologies are fast-developing and have been automating the entire chemistry research process in recent years. As a central science, chemistry should actively reach for these new technologies and search for new opportunities of cross-linking.

**LS Zheng:** AI and other technologies are changing chemistry and chemical engineering in not only scientific research but also industrial production. I also noticed that many young students are very interested and talented in these interdisciplinary fields. As educators, we should provide platforms for them. The Chemistry Teaching Guidance Committee is considering setting up a new major that combines AI with chemistry and chemical engineering. But apparently, there are many details to be designed and discussed.

How to improve the systems of choosing a major and the curricula is an old question for chemistry education. Many universities have made their own attempts. Some try to set up basic science classes which do not assign students to different majors in the first years. For chemistry courses, the curricular system has not been well established. The major courses, such as inorganic chemistry, physical chemistry and structural chemistry, are overlapping with each other. In recent years Xiamen University has been trying to perform a fundamental reform by breaking the

As a central science, chemistry should actively reach for these new technologies and search for new opportunities of cross-linking.

—Zhenfeng Xi

traditional system and re-organizing the chemical courses into several modules. In my opinion, reforms of the curricula system should be encouraged, but we should be very cautious about it for the reason that these reforms will directly influence students’ future development.

**J Pei:** We have many courses in the current higher chemistry education programs, but the courses are not organized systematically, and some courses overlap with each other. Moreover, the courses are often not deep enough and the students cannot learn much from them.

**LS Zheng:** That's true. With the development of chemistry, we always want to add more contents and set up more courses for students, but when we do so, we have to remove some content from the old courses, and the result is that class hours for many courses are declining.

Another point about the curricular system is that we should not set up a single uniform system for all the universities and colleges. They should have their own systems according to their levels and characteristics.

**Y Li:** I agree that the curricular systems should be differentiated. For example, universities began to enroll students under the Pilot Reform Program of Enrollment for Basic Subjects in 2020, and we should set up special curricular plans for these students so they get a better education.

## BETTER TEXTBOOKS ARE WANTED

**LS Zheng:** Our chemistry textbooks have been lagging behind the development of chemical science for a long time. The knowledge in these textbooks has usually been discovered tens or even hundreds of years ago, and if the teachers do not provide additional information in the lectures, the students would be unable to learn about the discoveries of recent decades. The Chemistry Teaching Guidance Committee, as well as some universities, are encouraging professors in textbook compiling, but this is not an easy task. Prof. Pei participated in the compiling of the new Organic Chemistry textbook, which is of high quality and gives a good example.

**J Pei:** I led the compilation of the second half of the Basic Organic Chemistry textbook, which took us three years to complete. Textbook editing is really hard work, which requires us to search and organize lots of documents. If we want to renew the textbooks every two or three years, it would be even harder and would require much more manpower and other resources. How to achieve these goals is a question we have to answer.

**S Gao:** Together with colleagues, I am trying to translate a general chemistry textbook written by James Anderson at Harvard University into Chinese. This is a well-written and well-organized textbook, which links the basic chemistry concepts with urgent human challenges such as the energy and environmental crisis, so that the interests of the students can be well stimulated. We should undertake substantial efforts to write such high-quality textbooks.

**J Pei:** Another issue is that our textbooks in high schools and universities are disconnected, and even contradict each other. The knowledge system is not continuous, and teachers of different educational stages know almost nothing about the textbooks of other stages. This creates many difficulties for the students to learn and understand chemistry, and may reduce their interest.

**LS Zheng:** Besides the connection with high schools, higher education should also better connect with the postgraduate stages, and provide meaningful help for graduates’ future careers. For students who become researchers, we need to provide them with more experimental training; and for students who move into industry, a survey shows that these students lack knowledge about automatic control, which is useful in many chemical industries.

**YD Li:** I have another suggestion on textbooks. Maybe we can compile a set of textbooks for the undergraduate students of the non-chemistry majors. It should contain the major concepts of general chemistry, physical chemistry, organic chemistry and the other chemistry core courses, and be organized in a clear structure, so that after several terms of learning, the students of different science and engineering majors can gain a clear understanding of the framework of chemistry and be able to apply that knowledge for their future careers. Such a set of textbooks can also serve as a good reference for students in high school, as well as a standard for the graduate school entrance examination of different majors.

**LS Zheng:** We have been wishing to compile such textbooks since a decade ago, but it is difficult to gather a group of qualified teachers to do it. A possible solution is to invite some experienced professors who are about to retire or have just retired.

If we want to renew the textbooks every two or three years, it would be even harder and require much more input of manpower and other resources.

—Jian Pei

## EXPERIMENTAL SKILLS TRAINING

**ZF Xi:** Chemistry is an experimental science, but in our chemistry education, especially at the middle- and high-school stages, experimental training is relatively lacking. It has become even harder to do classroom experiments due to COVID. ‘Computer simulated experiments’ are being used to replace real experiments, but these ‘fake experiments’ cannot foster the students’ practical abilities.

**LS Zheng:** With considerations of cost and safety, many high-school classroom experiments are cancelled or simplified, and the remaining ones are mostly performed by the teachers, but not the students. This is not good news for chemistry education.

We hope that the students can not only learn to use this equipment, but also disassemble and re-assemble them in class, so that they can really understand the working mechanisms of the machines.

—Lan-Sun Zheng

In recent years, some college chemistry teachers, including Prof. Junrong Zheng at Peking University and myself, have been trying to simplify some of the equipment used in research and turn them into educational equipment that can be used in undergraduate experimental courses. We hope that the students can not only learn to use this equipment, but also disassemble and re-assemble them in class, so that they can really understand the working mechanisms of the machines. We hope that these ‘big toys’ can foster students’ interest in chemistry and prepare them for chemical research.

Prof. Junrong Zheng has turned the Raman spectrometer into educational equipment, and we, at Xiamen University, have simplified the mass spectrometer. I hope that many other pieces of equipment, such as the scanning tunneling microscope, electron microscope, nuclear magnetic resonance instrument, and X-ray diffractometer, can also be simplified and used in undergraduate classes. To achieve these goals, we need to cooperate and negotiate with the equipment companies about how to simplify the machines and reduce their costs. This is not an easy process, but I believe that educational equipment will be useful for many universities in China and abroad.

**Y Li:** It's extremely important to improve the manual dexterity of Chinese students, who as children usually lack such training and are somewhat afraid of manipulating a piece of equipment when they become graduate students. So if we can use educational equipment to achieve these goals in the undergraduate stage, it will be a great help for them.

## THE ROLE OF PROFESSORS IN IMPROVING TEACHING QUALITY

**LS Zheng:** Our teaching quality needs to be improved. Especially in recent years, influenced by the pandemic, some courses have to be given online, while the effect of online courses is not satisfactory.

**J Pei:** Classroom teaching is a communication process between a teacher and students, while online courses cannot support such communication, it may act as an auxiliary measure, but cannot be the mainstream.

Moreover, the teaching quality of the regular offline courses is also not good enough. Many students learn only about the knowledge points—same as what they do in high-school; and the result is that they can pass the exams but their abilities to read, to comprehend and to actively expand their knowledge boundary are unimproved. Such education is not enough for college students.

**S Gao:** The aim of education is not only to teach knowledge and skills, but also to cultivate students into fully-developed adults with abilities of self-learning, independent-thinking and manual-practicing. Recently, a research published in *Nature Human Behavior* claims that after four years’ college education, the average critical thinking ability of Chinese students is not improved, but decreased—our education system may be a failure in some aspects.

**Y Li:** We can improve the students’ abilities in several aspects during chemistry courses. First, the ability to simplify and solve problems. Physics pursues accuracy, but chemistry often needs to solve practical problems in a more complex system, which means we have to identify the key points and rationally simplify the problems. If we can convey this thinking mode to students, it will be helpful for their future careers. Second, the ability of logical analysis. Students should understand that for every result, there must be a cause, and learn how to reason and analyse. Third, the ability of manual practice. And fourth, the ability to cooperate, which can be improved by setting group assignments for them.

Physics pursues accuracy, but chemistry often needs to solve practical problems in a more complex system.

—Yan Li

**LS Zheng:** That's right, and to improve those abilities, we need wonderful teachers. I think a good teacher for higher chemistry education should satisfy three requirements: they should have some years of educational experience; they should be familiar with the research frontiers; and they should be passionate for teaching.

The number of college teachers has grown quickly in recent years, but unfortunately, teachers meeting the abovementioned three requirements are far from enough. In some top universities, young teachers have chances to learn from the experienced teachers. But in many local colleges, young teachers, sometimes having just gained their PhD degrees and have not received any training on teaching, have to give lectures to students. Apparently, their teaching quality cannot be satisfactory.

Years ago, the National Natural Foundation of China organized several training programs for teachers in mid-west China. These programs provided chances for the local teachers to communicate with experienced teachers from top universities and learn about the modern teaching ideas and skills. We should organize more similar programs in the future.

**J Pei:** Few teachers are good at teaching, and even fewer teachers are willing to think about how to improve chemistry education. Maybe we should rebuild and improve the Teaching Research Offices in colleges, which can provide a platform for teachers to communicate and discuss about how to compile textbooks, how to reform curricular system and how to improve teaching quality.

## THE TRAINING OF GRADUATE STUDENTS

**S Gao:** Our last topic today is: how to better train the chemistry-majored graduate students?

**YD Li:** In recent years, Chinese universities have been enrolling many more graduate students than before. Especially, many local universities are beginning to cultivate graduate students, but in some of these universities, mentors are not qualified enough to foster innovative future chemists. So I think different universities should have different graduate educational goals and methods—some universities should focus on training future scientists, while others may train graduate students to become industrial talents that are able to solve practical problems.

**LS Zheng:** I agree that the universities should find their own ways of graduate education. I also observed that as more graduate students are enrolled, the mastery level of basic chemistry knowledge and skills of the postgraduate freshmen differ greatly. It means that we have to rearrange graduate students’ cultivation plans and help some of the freshmen to catch up.

**Y Li:** I noticed that many current graduate students are not really interested in scientific research. When encountering difficulties in their projects, many of them would not actively search for the solutions, and in that case, there would be little that the mentors could do to help.

**YD Li:** That may be a result of the changing environment. In the past, if a graduate student works hard on his or her project, gets good results, and masters professional skills, he or she is very likely to get a position in academia after graduation. But now, vacant positions in the academia are becoming fewer and fewer, many graduate students feel that even if they work hard, they are unlikely to become research scientists. Thus, they would lose their inner-motivation.

As mentors, what we can do is to help the students to set up realistic goals according to their specific personalities. The goals can be becoming chemists, scientists in interdisciplinary fields, engineers, or any other possible choices. With such realistic goals, students will be motivated to work hard for their own futures.

As mentors, what we can do is to help the students to set up realistic goals according to their specific personalities.

—Yadong Li

**J Pei**: That's right. In the past, the only goal of graduate student cultivation was to foster future scientists. But now, the cultivation goals should be diversified so that the graduates would become talents in various job positions that need chemistry knowledge and skills.

**S Gao:** Thank you for joining today's discussion. We hope that more chemistry teachers and researchers will pay more attention and devote more effort to the improvement of chemistry education.

